# A Comparative Evaluation of the Photosensitizing Efficiency of Porphyrins, Chlorins and Isobacteriochlorins toward Melanoma Cancer Cells

**DOI:** 10.3390/molecules28124716

**Published:** 2023-06-12

**Authors:** Kelly A. D. F. Castro, Nuno M. M. Moura, Mário M. Q. Simões, Mariana M. Q. Mesquita, Loyanne C. B. Ramos, Juliana C. Biazzotto, José A. S. Cavaleiro, M. Amparo F. Faustino, Maria Graça P. M. S. Neves, Roberto S. da Silva

**Affiliations:** 1 Department of Biomolecular Sciences, Faculty of Pharmaceutical Sciences of Ribeirão Preto, University of São Paulo, São Paulo 05508-220, Brazil; 2 LAQV-REQUIMTE, Department of Chemistry, University of Aveiro, 3810-193 Aveiro, Portugal

**Keywords:** isobacteriochlorin, chlorin, porphyrin, photodynamic therapy, photosensitizer, melanoma, skin cancer

## Abstract

Skin cancer is one of the cancers that registers the highest number of new cases annually. Among all forms of skin cancer, melanoma is the most invasive and deadliest. The resistance of this form of cancer to conventional treatments has led to the employment of alternative/complementary therapeutic approaches. Photodynamic therapy (PDT) appears to be a promising alternative to overcome the resistance of melanoma to conventional therapies. PDT is a non-invasive therapeutic procedure in which highly reactive oxygen species (ROS) are generated upon excitation of a photosensitizer (PS) when subjected to visible light of an adequate wavelength, resulting in the death of cancer cells. In this work, inspired by the efficacy of tetrapyrrolic macrocycles to act as PS against tumor cells, we report the photophysical characterization and biological assays of isobacteriochlorins and their corresponding chlorins and porphyrins against melanoma cancer cells through a photodynamic process. The non-tumoral L929 fibroblast murine cell line was used as the control. The results show that the choice of adequate tetrapyrrolic macrocycle-based PS can be modulated to improve the performance of PDT.

## 1. Introduction

Worldwide, the cancer incidence rate has increased, being one of the main causes of death. Currently, some means of treating cancer include surgery in the case of localized solid tumors, and radiotherapy and chemotherapy (for non-localized tumors) [1]. The development of new therapeutic agents that are selective, efficient, and cause minimal damage to the patient has become one of the central challenges of medicinal chemistry. Melanoma is a type of skin cancer that originates in melanocytes [2], which are found in the basal layer of the epidermis [2,3]. The resistance of this form of cancer to conventional treatments has led to the employment of some alternatives [3].

Photodynamic therapy (PDT) is a therapeutic methodology that requires the combination of a photosensitizer (PS), dioxygen (^3^O_2_), and light which, under specific conditions, generate reactive oxygen species (ROS) that are highly cytotoxic, inducing a reduction in the viability of cancer cells [1,4,5,6]. This is a therapeutic approach that has been pointed out by the scientific community as a promising complement or alternative to the conventional anticancer approaches, such as surgery or radiotherapy. PDT is a non-invasive therapy with high selectivity for cancer cells and reduced side effects due to its controllability and high spatiotemporal approach.

Porphyrins and related compounds are known for their excellent performance as photosensitizers (PS) in PDT [7,8,9,10,11,12,13]. Currently, applied PS are often based on porphyrins, chlorins, and isobacteriochlorin-type derivatives due to their optical features, namely, their strong absorption in the “therapeutic window” ranging from 650 to 850 nm, and their ability to produce ROS that can be lethal to the undesired tissue when activated by light [14,15,16,17,18]. Porphyrins and their derivatives that contain fluorine atoms in their structures have been investigated for more than 20 years [19,20,21,22,23,24]. The presence of fluorine atoms aims to modulate the pharmacokinetic and photophysical features, such as photostability, ROS production, and lipophilicity, among others. On the other hand, chlorin-based macrocycles exhibit photophysical properties similar to porphyrins, but with intensified and red-shifted Q bands, making chlorins the most promising candidates for PDT [4,17,25,26]. Like chlorins, isobacteriochlorins are also porphyrin-based reduced forms, but due to the presence of two reduced pyrrolic units in adjacent positions, they are more prone to oxidative processes. Synthetic strategies, such as the presence of bulky substituents next to the reduced pyrrolic-type unit or the modification of the porphyrinic core with exocyclic-fused rings, are usually used to avoid these oxidative processes [27,28].

Natural chlorins such as chlorophylls, which play a vital role in photosynthetic processes, are considered promising PS. However, the use of these naturally reduced porphyrins in PDT requires laborious extraction and modification of natural derivatives or total synthesis approaches, which are both a tremendous disadvantage [29,30]. To overcome this issue, efficient synthetic approaches to prepare chlorin-type macrocycles and other *meso*-tetraarylporphyrin reduced derivatives (e.g., (iso)bacteriochlorins) were developed, namely, those involving porphyrin precursors in cycloaddition reactions, such as Diels–Alder reactions and 1,3-dipolar cycloadditions [31].

We have recently studied the efficiency of 5,10,15-tris(pentafluorophenyl)-20-(4-pyridyl)porphyrin (**Por2**) and of the corresponding chlorin (**Chl2**) and isobacteriochlorin (**Iso2**) toward the B16F10 melanotic cell line. The **Chl2** and **Iso2** display a PDT effect and can induce a decrease of up to ca. 90% in the viability of the resistant B16F10 cell line after short irradiation periods with a maximum total light dose of 5.4 J.cm^−2^. In this study, it was found that the light irradiation on the therapeutic window (PDT effect) was strongly dependent on different factors, namely, the PS structure, ^1^O_2_-generation ability, PS cell uptake, and subcellular localization [32]. Additionally, porphyrin **Por1** and its reduced derivatives (**Chl1** and **Iso1**) were evaluated as PS toward prostate cancer PC-3 cells after incorporation into polyvinylpyrrolidone (PVP) formulations. The authors used this strategy to prevent aggregation issues and found that PS-PVP formulations bearing the porphyrin-reduced derivatives (**Chl1** and **Iso1**) display apoptosis-mediated PDT activity when irradiated with a red light and a total light dose of 10.6 J.cm^−2^ [33]. Drain and co-workers prepared a series of thioglycosylated compounds similar to **Por1**, **Chl1**, and **Iso1** and evaluated them as diagnostic agents. The uptake for such derivatives was evaluated into MDA-MB-231 breast cancer and K:Molv NIH 3T3 mouse fibroblasts, showing that the uptake at a nanomolar range of the thioglycosylated bacteriochlorin derivative makes it appropriate for tagging applications, while the chlorin analog is suitable for both targeting and treating diseased tissues due to its high absorption into the near-infrared region [34]. Later, Samaroo and co-workers [35] evaluated the interaction of those photosensitizers in the presence of plasma proteins, bovine serum albumin (BSA), and human serum albumin (HSA) through spectrophotometric and spectrofluorimetric titrations and theoretical studies. The interaction with the protein’s hemin site by porphyrinic-based PS leads to the formation of stable complexes, showing the potential of those proteins to be used as effective drug-delivery systems for further therapeutic purposes.

In the present work, we decided to evaluate and compare how the efficiency of isobacteriochlorin, chlorin, and porphyrin analogues against melanoma cancer cells would be affected by the type of *meso*-substituted scaffold selected—A_4_-type versus A_3_B-type. For this, **Por1**, **Chl1**, and **Iso1** were chosen as examples of *meso*-substituted A_4_-type macrocycles while for the A_3_B-type series, **Por2**, **Chl2**, and **Iso2** were selected. To investigate that influence, the photophysical/photochemical and biological features of both *meso*-substituted series A_4_-type and A_3_B-type derivatives were performed. The carried-out assays allowed us to compare how the photophysical and biological properties of the studied derivatives are modulated by inducing changes in one of the substituents at the *meso*-position. This comparative study can be a driving force for further studies using both the PS design and PDT assays as targets.

## 2. Results and Discussion

### 2.1. Photosensitizers: Synthesis and Characterization

**Figure 1:**
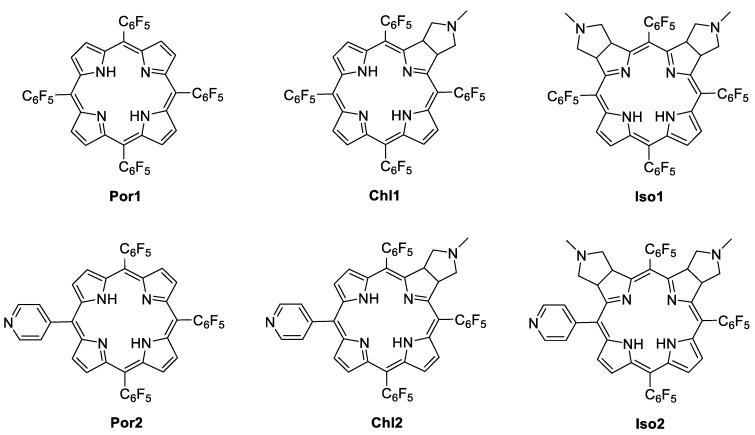
Structures of porphyrins, chlorins, and isobacteriochlorins studied in this work.

5,10,15,20-Tetrakis(pentafluorophenyl)porphyrin (**Por1**) and 5,10,15-tris(pentafluorophenyl)-20-(4-pyridyl)porphyrin (**Por2**) were obtained directly by condensation of pyrrole with the adequate aldehydes in acidic conditions, according to previously described procedures [32,36,37]. The pyrrolidine-fused chlorins (**Chl1** and **Chl2**) and isobacteriochlorins (**Iso1** and **Iso2**) were prepared throughout the 1,3-dipolar cycloaddition of the appropriate porphyrin (**Por1** or **Por2**) and the azomethine ylide generated from *N*-methylglycine and paraformaldehyde (Figure 1). The reduced derivatives were attained in yields similar to those reported in the literature (70% for **Chl1** and 49% for **Chl2**; 18% for **Iso1** and 15% for **Iso2**). The structures of all the compounds were confirmed by ^1^H and ^19^F NMR, UV–Vis spectroscopy, and mass spectrometry and agreed with the data reported in the literature [32,33,37,38,39,40].

The absorption, emission, and excitation spectra of porphyrins (**Por1,2**) and their reduced analogs, chlorins (**Chl1,2**), and isobacteriochlorins (**Iso1,2**), were acquired in *N*,*N*-dimethylformamide (DMF) and are summarized in Table 1. The photophysical characterization of **Por1** and **Por2** was assessed since they are the chemical precursors of the reduced derivatives **Chl1**, **Chl2**, **Iso1**, and **Iso2**. The absorption spectra of **Por1** and **Por2** exhibit the typical features of free-base *meso*-substituted porphyrin derivatives [41], displaying an intense Soret band at 410 and 412 nm, respectively, due to S_0_→S_2_ transitions, followed by four weak Q bands in the visible region ranging from 504 to 638 nm, attributed to the S_0_→S_1_ transitions. The Soret band on the absorption spectra of chlorins (**Chl1,2**) was slightly blue-shifted (*ca*. 5 nm) when compared with the corresponding porphyrin precursor (Figure S1). Concerning the Q bands region, significant red shifts (~14 nm) were observed for the two bands at higher wavelengths.

The UV–Vis spectrum of both isobacteriochlorins **Iso1** and **Iso2** shows Soret bands even more blue-shifted (20–25 nm) than those of the corresponding chlorin derivatives **Chl1** and **Chl2**; the last Q bands also show significant red shifts (10–15 nm) when compared with porphyrins. This red shift in the last Q band for both reduced series is of high interest for PDT, since this transition absorbs in the so-called therapeutic window (600–800 nm). As expected, **Chl1** and **Chl2** display the last Q band at ca. 650 nm with a relatively high intensity when compared with the other Q bands. The pyridyl group at the *meso*-position of the porphyrin macrocycle did not induce noticeable changes in the absorption features of the free-base porphyrin and reduced derivatives.

Concerning the steady-state emission spectra, **Por1**, **Por 2**, and **Iso2** showed similar emission spectra with two well-defined bands at 638 and ~700 nm. The **Iso1** emission spectrum displayed a similar profile; however, the first vibrational mode of the fluorescence was blue-shifted 38 nm to 600 nm. Both chlorin derivatives **Chl1** and **Chl2** only had one emission band at 654 and 649 nm, respectively (Figure S2). The emission spectra of the studied compounds are typical of free-base *meso*-tetraarylporphyrins and of their reduced derivatives [42,43,44].

The fluorescence quantum yields (Φ_F_) (Table 1) were determined in DMF using *meso*-tetraphenylporphyrin (**TPP**) as the standard (Φ_F_ = 0.11 in DMF) [45,46]. The fluorescence properties of each compound type appeared quite similar, which is consistent with the similarity of their chemical structure. The higher fluorescence quantum yields observed for **Chl1**, **Chl2**, **Iso1**, and **Iso2**, if compared to **Por1** and **Por2**, can be ascribed to the differences in their electronic structures and molecular geometries. Chlorins and isobacteriochlorin generally have a more distorted and non-planar structure compared to porphyrins [47]. This distortion can lead to a decrease in the non-radiative relaxation pathways, such as vibrational relaxation and internal conversion, thereby enhancing the fluorescence efficiency. Compound **Iso2** shows the highest fluorescence quantum yield (Φ_F_ = 0.21), followed by **Chl2** (Φ_F_ = 0.16), **Chl1** (Φ_F_ = 0.15), and finally, **Iso1** (Φ_F_ = 0.13). In fact, all the isobacteriochlorin and chlorin-type derivatives presented a greater fluorescence quantum yield (Φ_F_) than **TPP** (0.11). However, opposite behavior was observed for the starting porphyrins **Por1** and **Por2**, which showed a lower fluorescence quantum yield (Φ_F_) than **TPP** (respectively, 0.01 and 0.06 versus 0.11). The fluorescence lifetime of a singlet state (τ) found for porphyrins varied between 10 ns and 11.1 ns. The lifetime reduction observed for **Chl1** (6.02 ns) and **Chl2** (6.90 ns) when compared to **Por1** and **Por2** can be explained by the presence of a reduced pyrrole-type ring, leading to a reduction in the π-conjugation effect and improving the macrocycle distortion in the excited state, thus resulting in a decrease in radiative decay rates [48]. The emission decay profile for **Iso1** and **Iso2** derivatives can be fitted (Figure S3) by two decay components. For **Iso1**, we have 5.4 ns (~97%) and 1.2 ns (~3%), whereas the short-lived component for **Iso2** had a contribution of 77%, and the contribution of the long-lived component was lower when compared to **Iso1**. The reduction observed for the fluorescence lifetimes of isobacteriochlorins **Iso1** and I**so2** can be ascribed to the presence of an additional reduced pyrrolic-type ring at the macrocycle core. The increase in the number of reduced pyrrolic units conducts, in general, to an increase in the conformational flexibility (lower rigidity) of the macrocycle, affecting the electronic structure. Pyrrolidine-type units should decrease the π-conjugation effect, and they may improve the macrocycle distortion in the excited state, contributing to the reduction observed for the fluorescence lifetimes of isobacteriochlorins **Iso1** and **Iso2** and of chlorins **Chl1** and **Ch2**, leading to a fast internal conversion.

The long-lived triplet state’s (τ_T_) profile decay of the studied compounds range between 0.50 and 0.98 μs. For **Por2**, bearing a pyridyl group at one of the *meso*-positions, the value of the triplet lifetime is lower than the one displayed by **Por1**, with no pyridyl substituent (0.76 versus 0.98 μs). However, for the reduced derivatives, a different behavior is observed. **Chl2** (0.74 μs) and **Iso2** (0.63 μs) display higher triplet excited state lifetime values when compared with the corresponding analogs **Chl1** (0.51 μs) and **Iso1** (0.50 μs). The remarkable dependence of the triplet lifetime as a function of the substituent and its position is well established in the literature [44,49,50].

**Figure 2:**
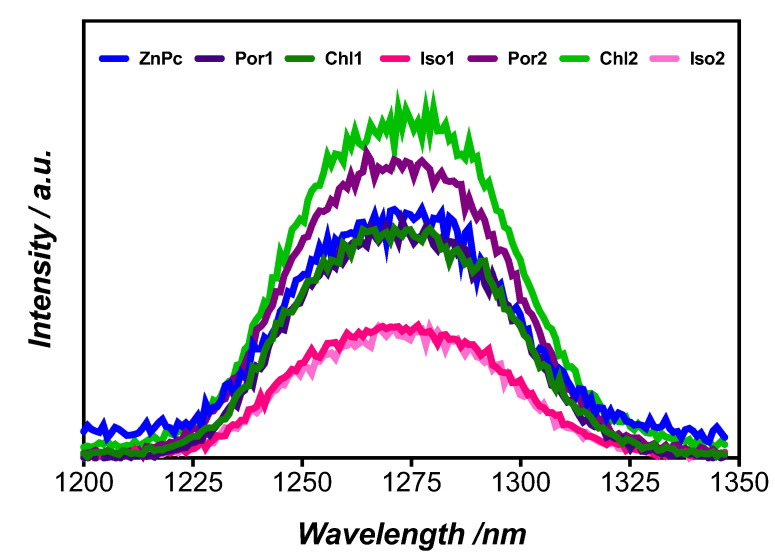
Near IR emission spectra of singlet oxygen produced by **ZnPc**, **Por1**, **Chl1**, **Iso1**, **Por2**, **Chl2**, and **Iso2** in DMF (λ_exc_ = 660 nm) with an OD = 0.1.

The efficiency of a PS in PDT is also related to its ability to generate reactive oxygen species (ROS), mainly singlet oxygen (^1^O_2_). ROS acts as signaling molecules, but they can also promote cellular damage by rapidly oxidizing cellular components. The measurement of the ^1^O_2_ quantum yield (Φ_Δ_) was assessed by the luminescence method, measuring the ^1^O_2_ phosphorescence at 1270 nm (Figure 2) and using **ZnPc** (zinc(II) phthalocyanine) as the reference. It is worth noting that this approach is not dependent on the dye concentration but only on the number of photons absorbed [51]. In general, the Φ_Δ_ increased when the pyridyl group was attached to the macrocycle backbone (except for **Iso2**) in the following order: **Chl2** (0.81) > **Por2** (0.65) > **Por1** (0.55) > **Chl1** (0.42) > **Iso2** (0.35) > **Iso1** (0.31) (Table 1). Additionally, the triplet lifetime (τ_T_) increased for chlorin-type derivatives as a function of the substituent, which consequently increased the Φ_Δ_ [52]_._ The presence of halogens influences the ability to generate ^1^O_2_ [53], photostability, and photophysical features [16,19]. The differences observed for the Φ_Δ_ are probably related to the macrocycle planar distortion in **Por1**, **Chl1**, and **Iso1** derivatives. The presence of halogen atoms leads to an improved intersystem crossing from the photosensitizer´s excited singlet and triplet states [53]; however, for the evaluated compounds, the presence of more halogen atoms did not favor ^1^O_2_ generation of **Por1** and its reduced analogs (**Chl1** and **Iso1**) when compared with those bearing a pyridyl unit (**Por2**, **Chl2**, and **Iso2**). This is undoubtedly related to the lower amount of T_1_ states quenched by ^3^O_2_ due to the occurrence of competing processes, reducing the formation of ^1^O_2_. Probably, different restrictions in the aryl ring rotations ascribed to the presence of three C_6_F_5_ and one pyridyl unit, higher asymmetry, and different electronic effects contributed to improve the ability of **Por2**, **Chl2**, and **Iso2** to generate ^1^O_2_ when compared with the corresponding **Por1** and reduced analogs **Chl1** and **Iso1** with four C_6_F_5_ substituents [54]. Singlet oxygen generation is strongly related to the probability of molecular dioxygen (^3^O_2_) colliding with the PS in the triplet state, which can be affected by steric effects, often associated with a decrease in the amount of the excited triplet state. Data in Table 1 agree with this and can be related to the presence of the pyrrolidine-fused rings on both reduced derivatives, resulting in a decrease in collision frequencies between the macrocycles and ^3^O_2_. The high Φ_Δ_ of **Chl2** seems to indicate higher success in the collisions involving ^3^O_2_ and this reduced derivative. The best ^1^O_2_ generator was **Chl2** (Φ_Δ_ = 0.81); however, all the compounds studied are able to generate this highly cytotoxic species (ROS), which makes them suitable to be used in PDT against cancer cells.

### 2.2. PDT Assays

**Figure 3:**
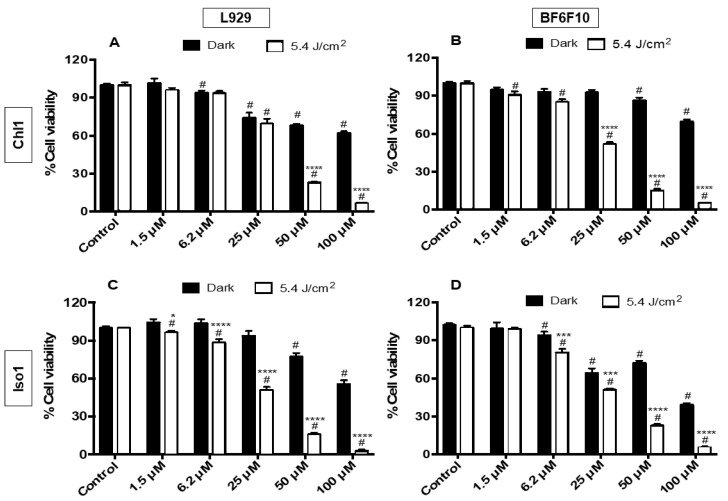
Cytotoxicity (dark) and photocytotoxicity (5.4 J/cm^2^) of **Chl1** (**A**,**B**) and **Iso1** (**C**,**D**) against L929 (**A**,**C**) and B16F10 (**B**,**D**) cells after irradiation (λ = 660 nm; light dose of 5.4 J/cm^2^). The results are presented as mean ± standard deviation. Significant differences relative to control cell cultures are presented with an ^#^ and relative to irradiated and non-irradiated by *. Statistical significance: ^#^ *p* < 0.05, * *p* < 0.05, *** *p* < 0.001, and **** *p* < 0.0001.

**Figure 4:**
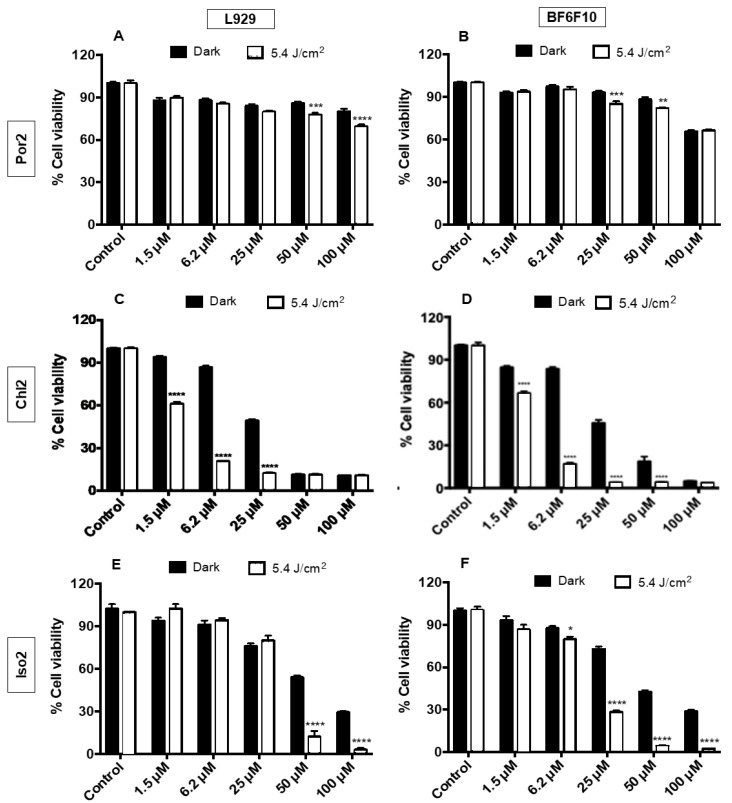
Cytotoxicity (dark) and photocytotoxicity (5.4 J/cm^2^) of **Por2** (**A**,**B**), **Chl2** (**C**,**D**), and **Iso2** (**E**,**F**) against L929 (**A**,**C**,**E**) and B16F10 (**B**,**D**,**F**) cells after red light irradiation (λ = 660 nm; light dose of 5.4 J/cm^2^). The results are presented as mean ± standard deviation. Significant differences relative to irradiated and non-irradiated by *. Statistical significance: * *p* < 0.05, ** *p* < 0.01; *** *p* < 0.001, and **** *p* < 0.0001.

With the exception of **Por1**, due to its low solubility in the RPMI/DMSO (1%) medium (vide infra), the ability of all the other derivatives **Chl1**, **Iso1**, **Por2**, **Chl2**, and **Iso2** to act as PS in the PDT assays was investigated, considering their efficiency to generate ^1^O_2_. The cytotoxicity of the compounds was assessed by an in vitro MTT colorimetric assay [3-(4,5-dimethylthiazol-2-yl)-2,5-diphenyl tetrazolium bromide] [55]. The viability of B16F10 murine melanoma cells was evaluated after treatment with **Chl1**, **Iso1**, **Por2**, **Chl2**, and **Iso2** at different concentrations (1.5, 6.2, 25, 50, and 100 μM), without and under red light irradiation (660 nm; 5.4 J/cm^2^) (Figure 3 and Figure 4). A non-tumoral fibroblast L929 murine cell line was used as the control.

The results show that in general, the compounds displayed cytotoxicity under non-irradiated conditions (dark) against both melanoma cells and non-tumoral cells at 25 μM or higher concentrations. However, in general, photo-stimulation increased their cytotoxicity. Of all the PS studied, globally speaking, **Chl1** was the least cytotoxic (Figure 3A,B) in the dark at 100 μM (38% and 31% cell death for L929 and B16F10, respectively). Both chlorins were effective toward B16F10 at 25 μM under red light irradiation (660 nm) with a reduction in the cell viability of 43% (**Chl1**) and 42% (**Chl2**), taking into account their toxicity under dark conditions (Figure 3B and Figure 4D). This phototoxicity improves at higher concentrations, attaining cell deaths of ca 64% at 100 μM, considering also the cytotoxicity under dark conditions. Despite the efficiency of both chlorins, these PS displayed small selectivity for B16F10 cells, causing the death of the non-tumoral cells ranging from 30% to 35% (Figure 3A and Figure 4C).

Although both isobacteriochlorins induced a reduction in tumoral cells, **Iso2** was more efficient and selective for B16F10 cells (*p* < 0.001) (Figure 4E,F) than **Iso1** (Figure 3C,D). For instance, **Iso1** at 25 μM displayed cytotoxicity under both dark (~36%) and light conditions (~49%) toward B16F10 cells and similar phototoxicity toward the non-tumoral cells (cell death of ~50%). Although there was less cytotoxicity for non-tumoral cell lines, the photocytotoxicity was more relevant than the cytotoxicity at higher concentrations. No improvement in the selectivity toward B16F10 cells was observed for isobacteriochlorin **Iso1**. Moreover, **Iso2** at 25 μM presented lower cytotoxicity in the dark and was able to cause 72% of cell death of the treated melanoma cells, and no significative cell death (20%) was achieved in non-tumoral cell lines under similar conditions.

Therefore, when comparing porphyrin and chlorin-type derivatives using the same concentration, chlorins seem to be more efficient PS after being irradiated with red light (660 nm) due to their strongest absorption in the red region. In general, the more photocytotoxic compounds contain the pyridyl group in their structure (**Chl2** and **Iso2**). In fact, there is a strong relationship between structure and activity. The efficiency of **Chl2** can be attributed to its photophysical and photochemical properties, mainly the production of ^1^O_2,_ but no selectivity for B16F10 cancer cells was observed; this PS was also able to induce a significant reduction in the non-tumoral fibroblast L929 murine cell line. Additionally, its dark activity can be due to the formation of adducts with biological structures that affect the cell survival [56], since the production of ROS is limited. Therefore, cellular uptake and cellular sublocalization should also be considered.

Several factors can influence the PS activity; the formation of aggregates is the feature that contributes the most to the decrease in its efficiency, due to the limitation of ROS production and cellular uptake [57,58]. Aggregation studies were performed in order to understand the potential effect of the porphyrin-type scaffold selected (*meso*-substituted—A_4_-type versus A_3_B type) on the cytotoxicity observed. UV–Vis studies assessing different biological PS concentrations in RPMI medium and DMSO (1%) were performed (see ESI, Figures S4–S8). It was noticed that the PS strictly followed the Beer–Lambert law to established concentrations, thus suggesting no aggregation in RPMI at concentrations below 3 μM for **Chl1**, 12.5 μM for **Iso1**, 6.0 μM for **Por2**, 3.0 μM for **Chl2**, and 3.0 μM for **Iso2**. **Por1** showed low solubility in biological conditions; so, it was decided not to evaluate this compound in the further PDT assays. The aggregation studies showed that the pyridyl group increased the PS solubility of **Por2**, but no noticeable changes were observed for **Chl2**, although leading to a decrease in the solubility of derivative **Iso2** when compared with the corresponding A_4_-type derivative.

As expected, more fluorine atoms increase the lipophilicity of the PS, which is corroborated by cellular uptake studies (Figure S9).

The octanol:water partition coefficients (Log *p*) were calculated for all the macrocycles using Molinspiration WebME Editor 3.81 [47]. The A_4_-type macrocycles exhibited Log *p* values between 9.98 and 9.75, while for the A_3_B-type macrocycles, the Log *p* values ranged from 9.69 to 9.39. Considering the possibility that at physiological pH the pyrrolidine units must be almost entirely in the cationic form (pyrrolidine pKa = 11.3), partition coefficients were also calculated for chlorin and isobacteriochlorin derivatives while taking that into account. A slight decrease was observed for Log *p* values of both series of protonated compounds, being 9.21 (**Chl1**) and 9.01 (**Iso1**) for A_4_-type reduced macrocycles and 8.60 and 7.93 for the corresponding A_3_B-type macrocycles **Chl2** and **Iso2**, respectively.

The uptake of **Chl1** and **Iso1** increased slightly as a function of concentration. On the other hand, both **Chl2** and **Iso2** showed a reduction in the uptake at 25 μM after 4 h of incubation in B16F10 cells evaluated by fluorescence; if compared to the concentrations of 6.1 μM and 12.5 μM, this behavior can be partially justified by the aggregation phenomena, corroborating the main findings in aggregation studies.

When the stability of the PS in RPMI/DMSO (1%) mixture was evaluated in the dark over 24 h (Figure S10), a noticeable decrease (~40%) in the stability was observed for **Chl1** and **Chl2**, and even after this long period in the solution, **Iso1** and **Por2** showed an acceptable stability, with a decrease of around 20%. **Iso2** showed the lowest stability in solution, with a decrease of around 45% in the absorption percentage of this PS after the same period. These data show that, under dark conditions, **Iso1** and **Por2** were the most stable in the RPMI/DMSO (1%) mixture at 3.1 μM after 24 h. When irradiated for 30 min under the same irradiation conditions of biological assays, the studied compounds showed good stability. Apart from **Chl2** (Figure S11D), which displays a noticeable decrease in the absorption intensity at Soret band, the UV–Vis spectra of the other compounds remained almost unchanged (Figure S11).

**Figure 5:**
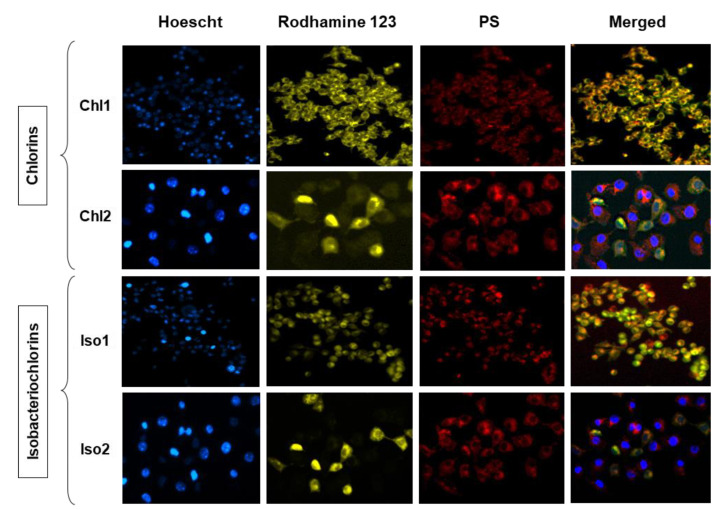
Fluorescence microscopy images of B16F10 cells treated with chlorins (**Chl1** and **Chl2**) and isobacteriochlorins (**Iso1** and **Iso2**). From left to right: blue fluorescence (Hoechst 33342), green fluorescence (Rhodamine 123), red fluorescence (**Chl1**, **Iso1**, **Chl2**, and **Iso2**) and merged images.

To demonstrate the intracellular distribution in B16F10 cells, images of fluorescence microscopy were acquired. Red fluorescence was observed in all cases, which led us to suggest that the PS was efficiently internalized (Figure 5). The two panels on the left side displayed the PS internalized in the B16F10 cells, with Hoechst staining the nucleus (blue) and Rhodamine staining the mitochondria (green). Chlorins (**Chl1** and **Chl2**) and isobacteriochlorins (**Iso1** and **Iso2**) showed similar fluorescence patterns with the mitochondria probe. The overlapping fluorescence of **Chl2** and **Iso2** suggests that the compounds are preferentially localized in regions near mitochondria, while **Chl1** and **Iso1** are apparently spread throughout the cell, including the nucleus.

Despite the lack of selectivity for cancer cells (B16F10) for most of the tested PS at high concentrations, we propose to employ metronomic PDT to improve the tumor-specific response. Wilson et al. created the term metronomic PDT, which consists of administering both the PS and light during an extended period at very low doses and over many hours in order to increase the selective reduction on the viability of cancer cells by apoptosis. In their study, the authors aimed to compare standard or acute PDT with metronomic PDT. At the end, they found that metronomic PDT enhanced tumor-specific cell death, while decreasing the harm to adjacent normal tissues [59]. Therefore, herein we suggest that metronomic delivery or several PDT treatments are required to increase the selectivity of the PS we have tested, namely, of the chlorin-type derivatives.

## 3. Materials and Methods

### 3.1. Generalities

Absorption spectra were obtained on a Shimadzu UV-2501PC spectrophotometer in the 350–800 nm range. The fluorescence spectra were recorded in DMF in 1 cm × 1 cm quartz optical cells under normal atmospheric conditions on a computer-controlled F4500—Hitachi spectrofluorometer. The widths of both excitation and emission slits were set at 3.0 nm. To calculate the fluorescence quantum yield (Φ_F_), TPP in DMF was used as reference using Equation (1). In this equation, Φ_F_ is the fluorescence quantum yield of the sample; Φ_st_ is the fluorescence quantum yield of **TPP** (λ_exc_ = 420 nm, Φ_F_ = 0.11 in DMF); A_st_ is the absorbance of **TPP** and A is the absorbance of the sample at the excitation wavelength; S_st_ and S represent the integrated emission band of the **TPP** and sample, respectively.

Φ_F_ = Φ_F_ = Φ_st_ × S/S_st_ × A_st_/A (1)

Laser flash photolysis experiments for detection of the triplet state in solution were performed on a system using a Quanta Ray Lab-130 4 Hz Nd:YAG laser at 420 nm from Spectra Physics as the excitation source. The fluorescence lifetime was determined on a Fluorescence Correlation Spectroscopy/Fluorescence Lifetime Imaging coupled to a laser with 420 nm excitation wavelength. Direct measurement of singlet oxygen was performed by luminescence method from Equation (2), as described in the literature [60], using **ZnPc** as standard (λ_exc_ = 660 nm, Φ_Δ_ = 0.56 in DMF) [61,62].

Φ_Δ_ = Φ_*st*_ (I_s_/I_st_) (2)

where Φ_Δ_ is the singlet oxygen quantum yield of the sample; Φ_st_ the singlet oxygen quantum yield of **ZnPc**; I_st_ is the phosphorescence intensity of ^1^O_2_ at 1270 nm for **ZnPc**; and I_s_ the phosphorescence intensity for the sample.

### 3.2. Synthesis of the Photosensitizers (PS)

The photosensitizers were synthesized as described in the literature [32,36,37].

### 3.3. Cell Culture

The B16F10 (melanoma murine) and L929 (fibroblast murine) cells were obtained from the American Type Culture Collection. The cell line was cultured in RPMI medium with 10% supplemental fetal bovine fetal serum, 100 IU mL^−1^ of penicillin G, 100 mg mL^−1^ of streptomycin, and 1 µg mL^−1^ of amphotericin. Cells were seeded until 75–90% confluence in 96-well plates and cultured in a humidified incubator at 37 °C with 5.0% CO_2_ for 24 h.

### 3.4. Cell Viability Assay

Evaluation of cell cytotoxicity by porphyrin derivatives was performed against two different cell lines: the murine melanoma, B16F10, and murine fibroblast, L929. To this end, 2 × 10^4^ cells were incubated for 24 h in 96-well cell culture plates. After this period, the treatments with **Chl1**, **Iso1**, **Por2**, **Chl2**, and **Iso2** that were previously dissolved in DMSO (1.5, 6.2, 25, 50, and 100 μM), then dissolved in the culture medium, and serially diluted to the appropriate concentration to give a final DMSO concentration of 1%, were conducted with and without red light irradiation (emitted by an array of 96 light-emitting diodes (LEDs), λ = 660 nm). After the indicated treatment, the cells were incubated at 37 °C for 3 h in a culture medium containing 10 mol L^−1^ MTT in RPMI without supplemental fetal bovine fetal serum. The blue MTT formazan precipitate was then dissolved in 50 μL of DMSO, and the absorbance was measured at 480 nm with a multi-well plate reader. The cell viability was expressed as the percentage of the absorption values in the treated cells relative to the non-treated (control) cells. The data are presented as an average of three independent experiments with replicates.

### 3.5. Statistical Analysis

Statistical analysis was performed by two-way ANOVA. Equality of variance was assumed with Bonferroni’s post hoc test for pair-wise comparisons. Results with *p* < 0.05 were considered statistically significant.

### 3.6. Fluorescence Studies

Cellular uptake of porphyrin derivatives by B16F10 cells was performed by fluorescence spectroscopy and microscopy, as described in the literature [32]. The fluorescence was measured with a microplate reader (Spectra Max Paradigm) set at λ_exc_ = 420 nm, matching the Soret band and corresponding emission wavelength to each PS at the initial time = after 4 h of incubation. For fluorescence microscopy, specific organelles’ staining probes were used. Mitochondria were stained with Rhodamine 123, and Hoechst 33342 was used as the nuclei probe. After washing two times with PBS, the cells were examined by fluorescence microscopy (Nikon Eclipse Ti Microscope model TI-FL) using the following filters: DAPI (λ_exc_ at 340/380 nm and λ_em_ at 435 to 485 nm), FITC (λ_exc_ at 488 nm and λ_em_ at 505 to 527 nm), and Cy5 (λ_exc_ at 620/660 nm and λ_em_ at 662.5/737.5 nm) for porphyrin derivatives detection.

### 3.7. Stability Studies

The stability of each porphyrin derivative was verified by UV–Vis at regular intervals for up to 24 h under dark conditions. Photostability studies of the porphyrin derivatives were performed by irradiation of a solution of each derivative in RPMI and DMSO (1%) under the same conditions of PDT assays. The solutions were stirred and kept at ambient temperature.

## 4. Conclusions

In this work, two different series of neutral tetrapyrrolic macrocycle-based PS were successfully prepared and characterized. All the compounds are able to generate ROS, namely ^1^O_2_, which shows their potential to be used as PS in PDT against tumor cancer cells. However, due to the high aggregation observed for the A_4_-type **Por1** under the conditions where biological assays were carried out, the PS activity of this compound was not evaluated. So, the PDT efficiency of the A_4_-type reduced derivatives (**Chl1** and **Iso1)** as well as all the A_3_B-type porphyrinic PS prepared (**Por2**, **Chl2**, and **Iso2**) was evaluated toward murine melanoma cells (B16F10 cells) and non-tumor fibroblast murine cell lines (L929).

The cytotoxicity of the compounds evaluated is strongly dependent on their structure, level of reduction in the tetrapyrrolic macrocycle, and concentration. **Iso2** and **Chl2** were the most efficient PS against B16F10 cancer cells; however, **Chl2** also displayed higher (photo)cytotoxicity for non-tumor L929 cells.

Although both chlorins can be considered efficient PS against B16F10 cancer cells, they also showed high (photo)cytotoxicity for non-tumoral L929 cells. A different situation was observed with **Iso2**, whereby at 25 μM, it displayed good selectivity for B16F10 tumoral cells, being able to induce a cell viability reduction of 72% and only 20% of cell death of non-melanoma L929 cells; however, **Iso1**, with the same degree of reduction and efficiency to generate ^1^O_2_, was cytotoxic under both dark and light conditions. These results confirm how a simple manipulation of the porphyrin core can affect the photodynamic action of this type of reduced PS.

In sum, the results showed that the choice of an adequate tetrapyrrolic macrocycle can be modulated to improve the performance of PDT. The presence of a pyridyl unit affords tetrapyrrolic macrocycle-based PS with improved features to be used in PDT. Additionally, the PDT efficiency was highly dependent on the subcellular localization mechanism and cellular uptake.

This study is a pertinent contribution to both tetrapyrrolic macrocycle-based PS development and PDT fields, providing significant data to modulate the tetrapyrrolic macrocycle core and aiming to fine-tune its photophysical/photochemical properties. In the future, it will help the scientific community to design and better prepare PS with improved features and PDT activity to overcome the challenges of effectively reducing the viability of cancer cell lines.

## Figures and Tables

**Table 1:** Selected photophysical properties of porphyrins, chlorins, and isobacteriochlorins in DMF.

Compound	Soret Band λ_max_ (nm)	Q Bands λ_max_ (nm)				^a^ λ_emission_ (nm)	^b^ Φ_F_	^c^ τ_1_ (ns)	^c^ τ_2_ (ns)	^c^ τ_T_ (μs)	^d^ Φ_Δ_
**Por1**	410	504	539	582	638	638/698	0.01	10.0 (100)	-	0.98	0.55
**Chl1**	405	505	540	595	653	654	0.15	6.02 (100)	-	0.51	0.42
**Iso1**	380	510	548	586	648	600/654	0.13	5.40 (97)	1.20 (3.0)	0.50	0.31
**Por2**	412	506	538	582	635	638/700	0.06	11.1 (100)	-	0.76	0.65
**Chl2**	406	504	532	595	649	649	0.16	6.90 (100)	-	0.74	0.81
**Iso2**	386	506	540	580	660	638/700	0.21	6.39 (23)	1.58 (77)	0.63	0.35

(a) Optical density of all samples was 0.05 at 420 nm; (b) using **TPP** as a reference in DMF (Φ_F_ = 0.11); (c) λ_exc_ = 420 nm; (d) using **ZnPc** as a reference in DMF (Φ_Δ_ = 0.56) at 660 nm.

## Data Availability

The data that support the findings of this study are available from the corresponding authors upon reasonable request.

## Supplementary Materials

The following supporting information can be downloaded at https://www.mdpi.com/article/10.3390/molecules28124716/s1, Figure S1: UV–Vis spectra; Figure S2: Fluorescence spectra; Figure S3: emission decay fittings; Figures S4–S8: Aggregation studies; Figure S9: Cellular uptake; Figure S10: Stability; Figure S11: Photostability studies.

## Sample Availability

Samples of the compounds **Por1**, **Por2**, **Chl1**, **Chl2**, **Iso1**, and **Iso2** are available from the authors.
