# Supplementary material for: A Comparative Evaluation of the Photosensitizing Efficiency of Porphyrins, Chlorins and Isobacteriochlorins toward Melanoma Cancer Cells
Source: Molecules. 2023 Jun 12;28(12):4716. doi: 10.3390/molecules28124716 (PMC10300831; doi:10.3390/molecules28124716)
Supplement: Supplementary file 1 [file molecules-28-04716-s001.zip › molecules-2420286-supplementary.pdf]

Supporting Information

A comparative evaluation of the photosensitizing efficiency of porphyrins, chlorins and isobacteriochlorins towards melanoma cancer cells

Kelly A.D.F. Castro[1,2,*], Nuno M.M. Moura[2,*], Mário M.Q. Simões[2,*], Mariana M.Q. Mesquita[2], Loyanne C.B. Ramos[1], Juliana C. Biazzotto[1], José A.S. Cavaleiro[2], M. Amparo F. Faustino[2], Maria da Graça P.M.S. Neves[2] and Roberto S. da Silva[1]

[1] *Department of Biomolecular Sciences, Faculty of Pharmaceutical Sciences of Ribeirão Preto, University of São Paulo, SP, Brazil*

[2] *LAQV-REQUIMTE, Department of Chemistry, University of Aveiro, 3810-193 Aveiro, Portugal*

Index

UV-Vis spectra **2**

Fluorescence spectra **2**

Aggregation studies **4**

Cellular uptake **7**

Stability **8**

Photostability studies **9**

UV-Vis spectra

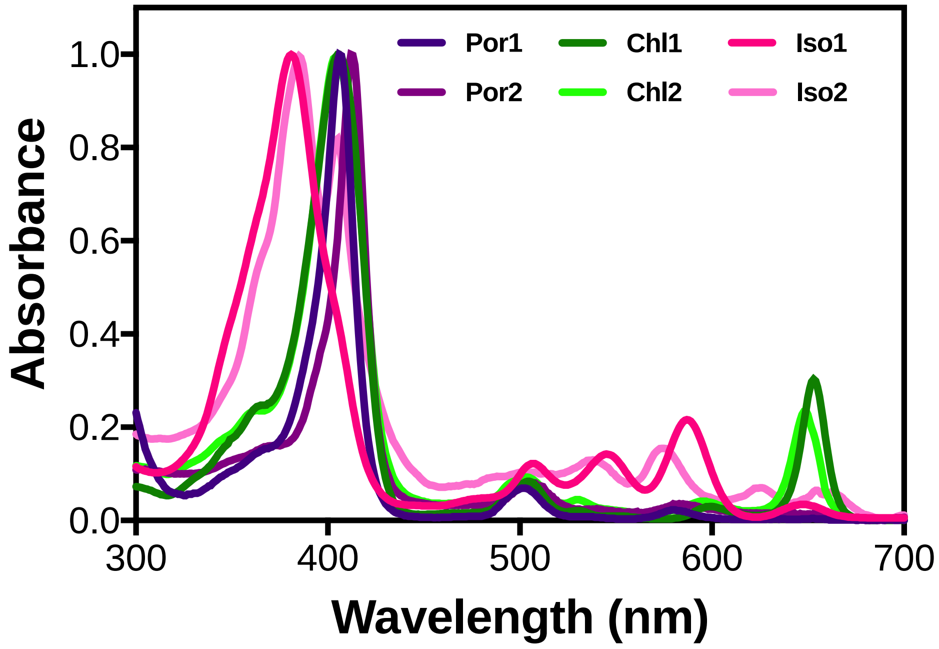

Figure S1. Normalized UV-Vis spectra of PS in DMF solutions.

Fluorescence spectra

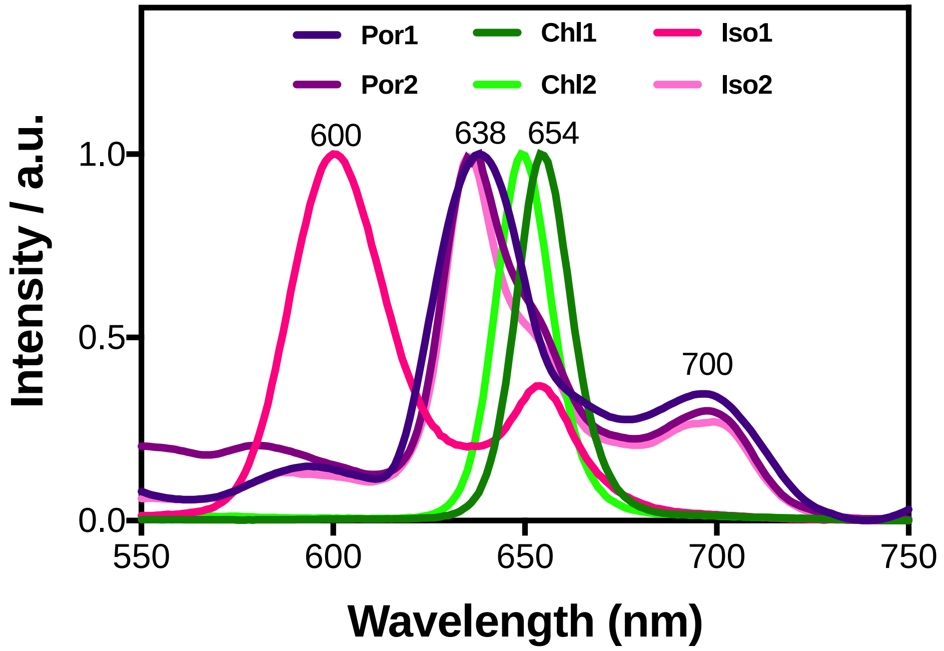

Figure S2. Normalized emission spectra of PS in DMF solutions, λ_{exc} = 420 nm.

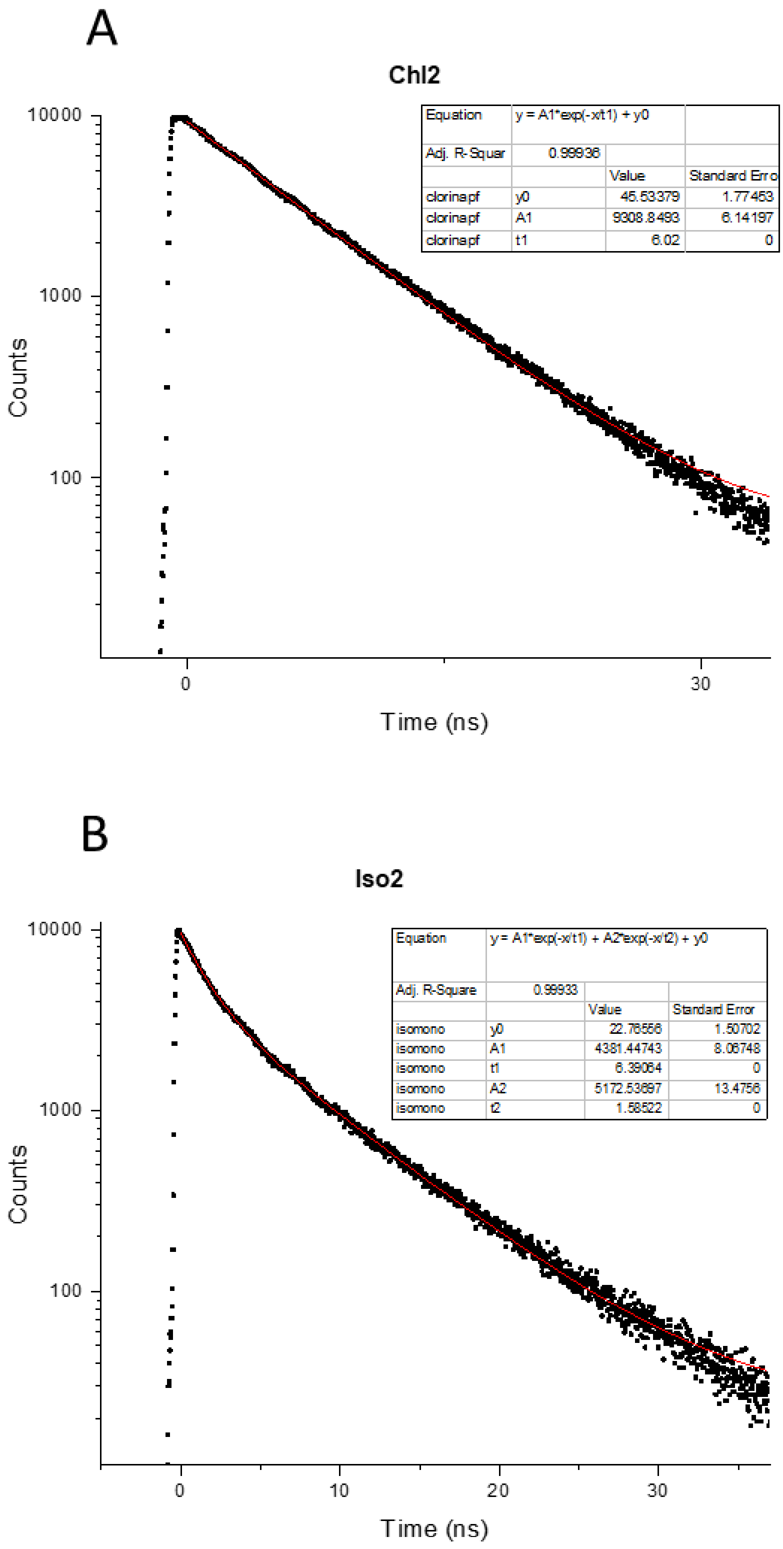

Figure S3. Chl2 (A) and **Iso2** (B) emission decay fittings.

Aggregation studies

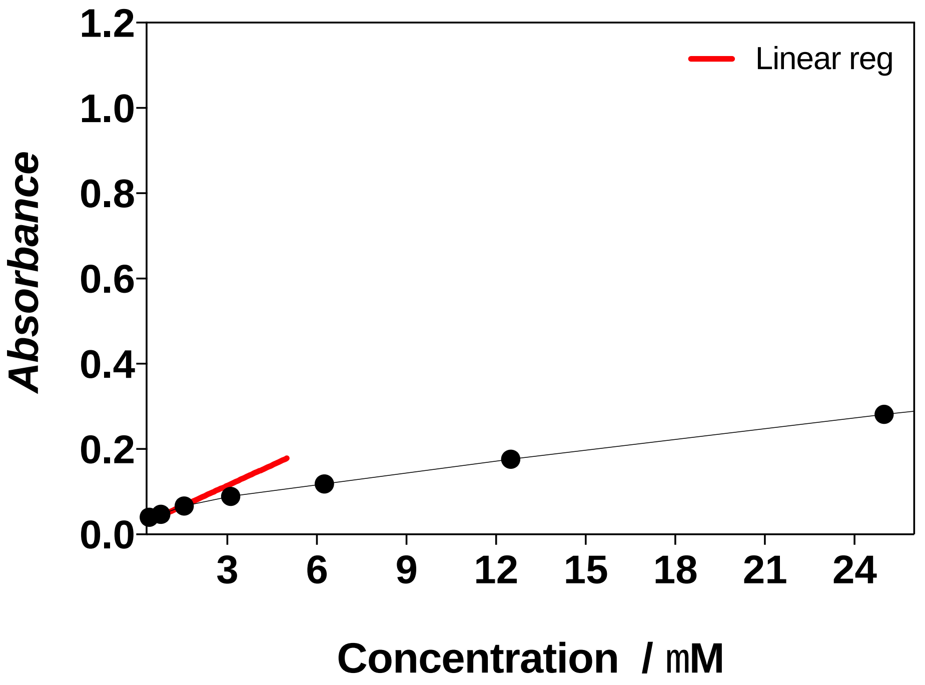

Figure S4. Plots of the Soret band at 405 nm *versus* concentration of **Chl1** in RPMI and DMSO (1%).

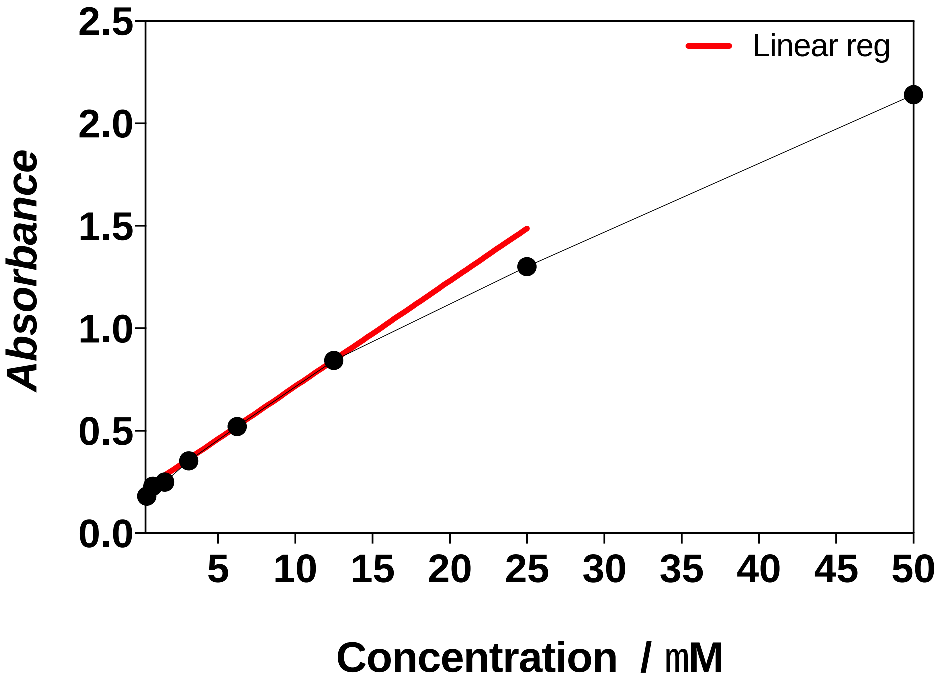

Figure S5. Plots of the Soret band at 380 nm *versus* concentration of **Iso1** in RPMI and DMSO (1%).

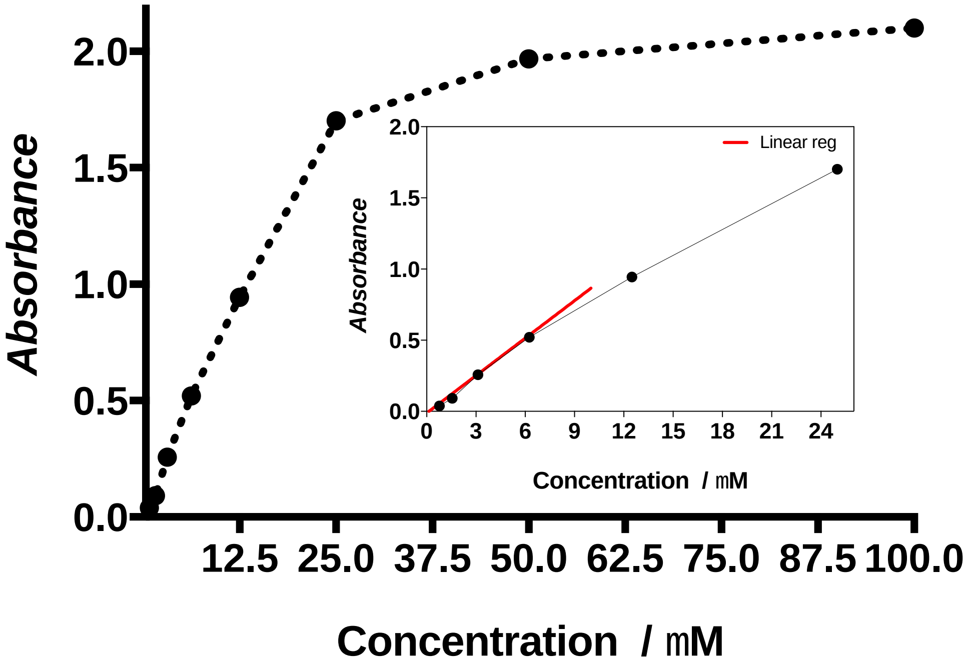

Figure S6. Plots of the Soret band at 412 nm *versus* concentration of **Por2** in RPMI and DMSO (1%). Inset: Linearity of the Soret band at 412 nm *versus* concentration of **Por2**.

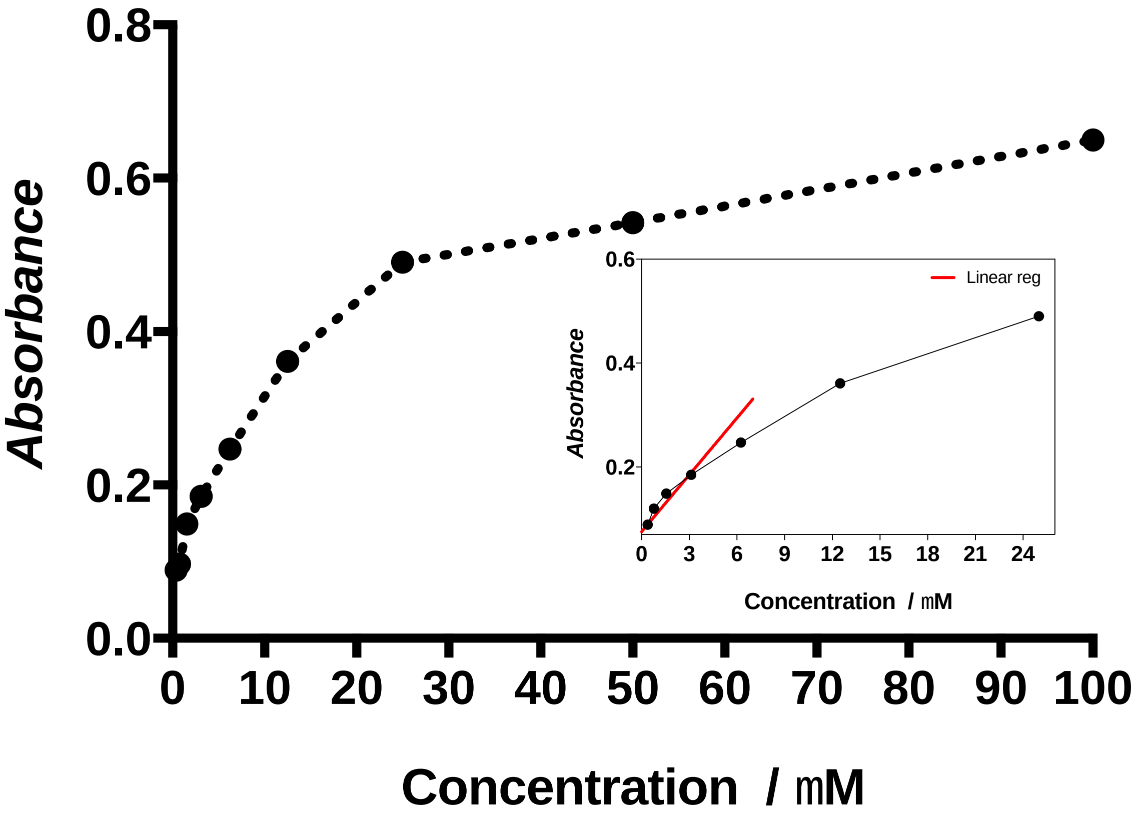

Figure S7. Plots of the Soret band at 406 nm *versus* concentration of **Chl2** in RPMI and DMSO (1%). Inset: Linearity of the Soret band at 406 nm *versus* concentration of **Chl2**.

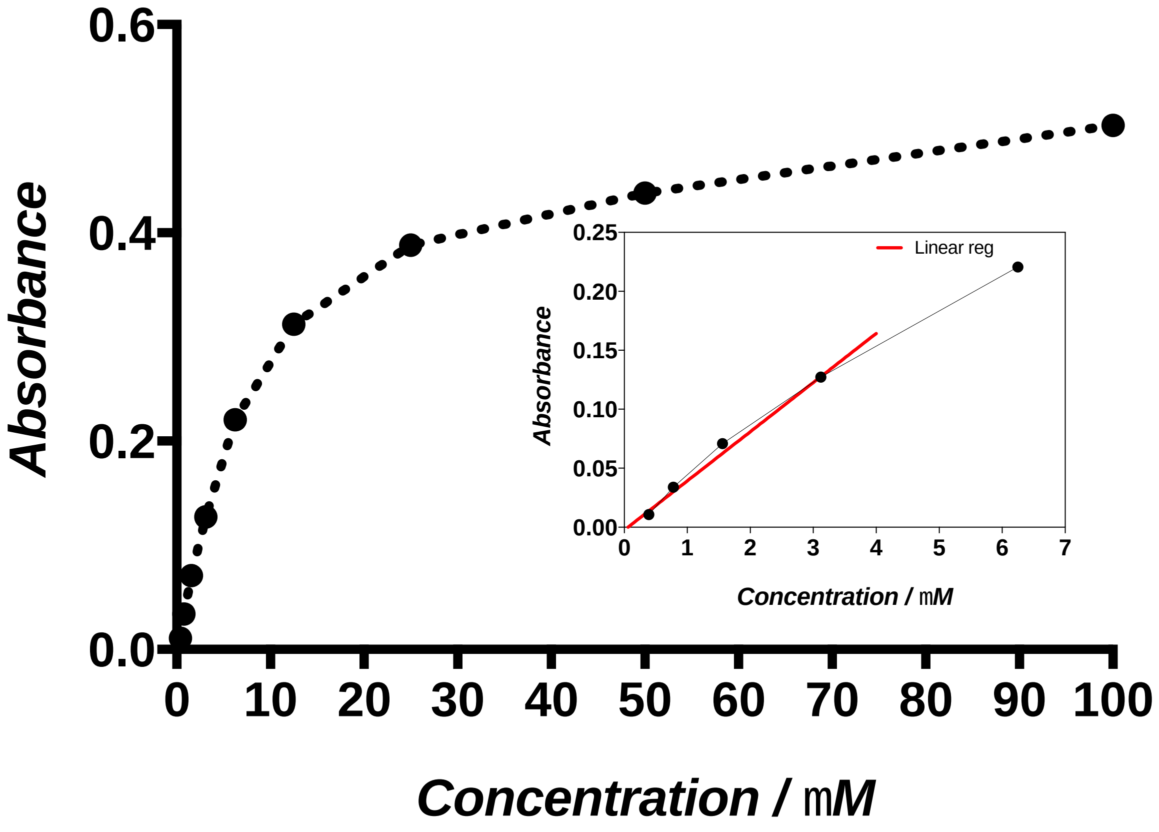

Figure S8. Plots of the Soret band at 386 nm *versus* concentration of **Iso2** in RPMI and DMSO (1%). Inset: Linearity of the Soret band at 386 nm *versus* concentration of **Iso2**.

Cellular uptake

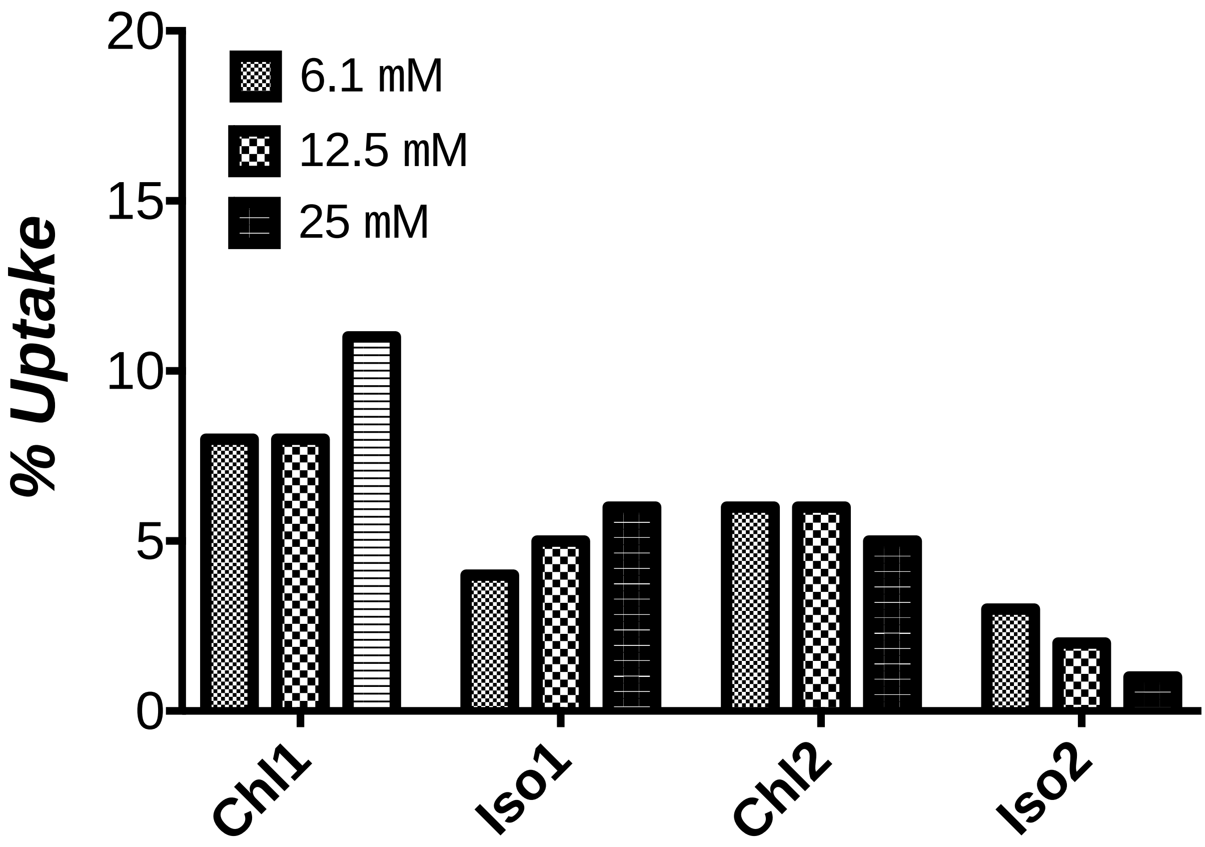

Figure S9. Quantitative cellular uptake in B16F10 cells after 4 h as a function of the photosensitizer concentration.

Stability

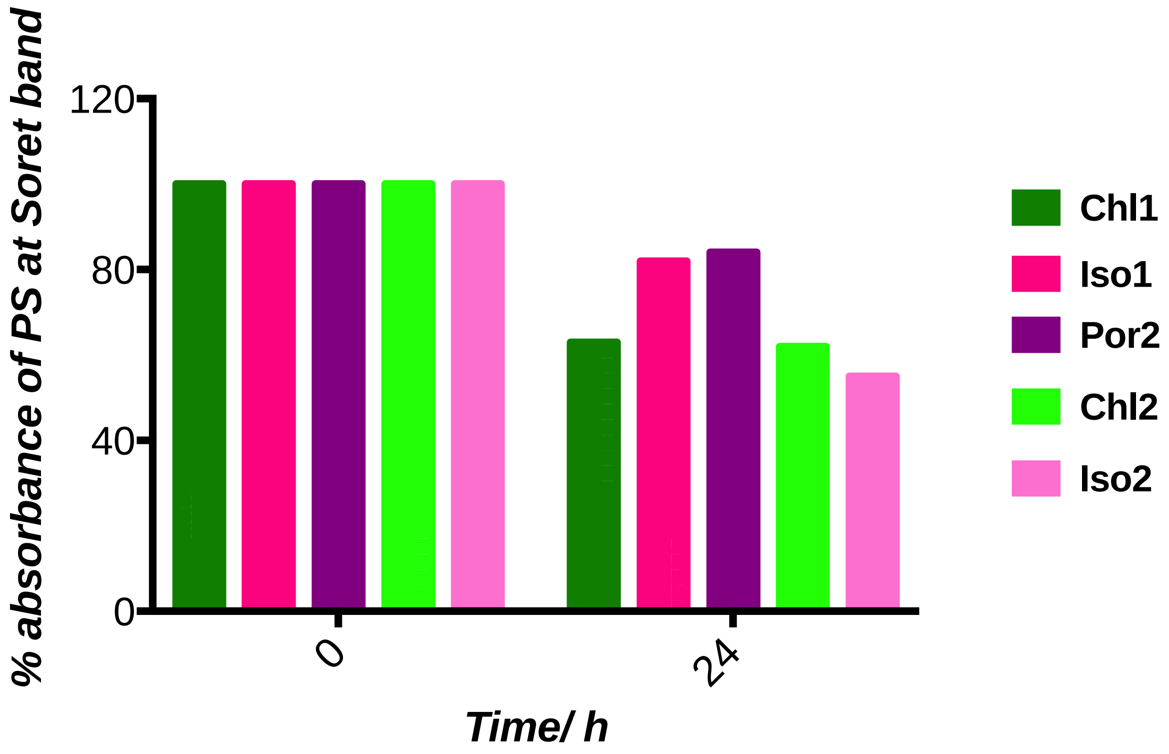

Figure S10. UV-Vis spectrophotometric study of **Chl1**, **Iso1**, **Por2**, **Chl2** and **Iso2** solution in RPMI/DMSO (1%) mixture at 3.1 μM under dark conditions, based on the Soret band intensity monitorization. The ordinate axis shows the percentage of PS in solution.

Photostability studies

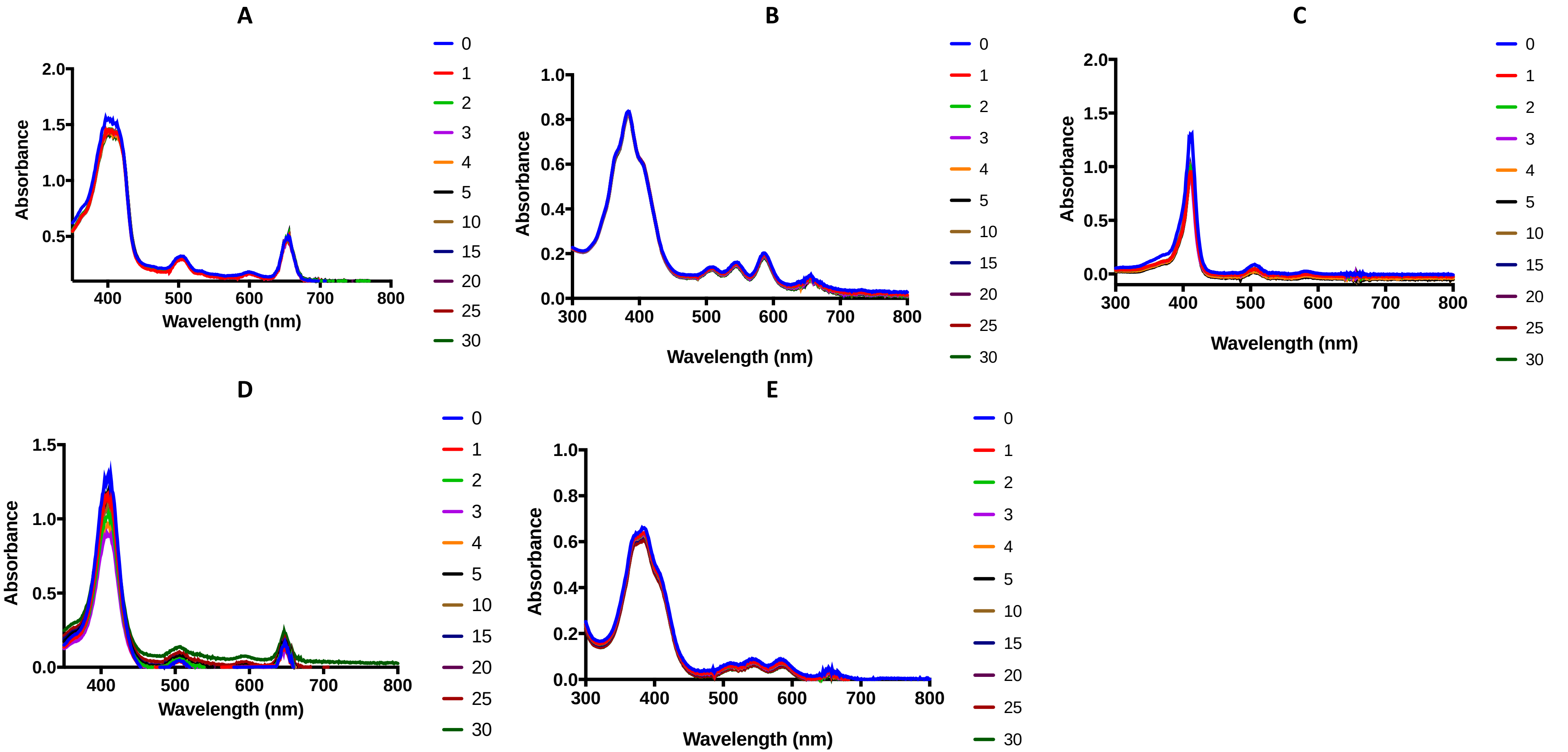

Figure S11. UV-Vis spectra of the various PS after irradiation at different times using the same conditions of PDT experiments. (A) **Chl1**, (B) **Iso1**, (C) **Por2**, (D) **Chl2** and (E) **Iso2**.
